# Morphologic and electrophysiologic findings of retinal degeneration after intravitreal sodium iodate injection following vitrectomy in canines

**DOI:** 10.1038/s41598-020-60579-1

**Published:** 2020-02-27

**Authors:** So Min Ahn, Jungryul Ahn, Seongkwang Cha, Cheolmin Yun, Tae Kwann Park, Young-Jin Kim, Yong Sook Goo, Seong-Woo Kim

**Affiliations:** 1 Department of Ophthalmology, Korea University College of Medicine, Seoul, Korea; 2 Department of Physiology, Chungbuk National University School of Medicine, Cheongju, Korea; 3 Department of Ophthalmology, Bucheon Hospital, Soonchunhyang University College of Medicine, Bucheon, Korea; 4 Medical Device Development Center, Osong Medical Innovation Foundation, Cheongju, Korea

**Keywords:** Retinal diseases, Experimental models of disease

## Abstract

We developed and characterized a canine model of outer retinal degeneration induced by sodium iodate (SI) intravitreal injection after vitrectomy. In the preliminary study, we repeatedly injected SI intravitreally into the eyes of three canines to develop outer retinal degeneration two weeks after vitrectomy. Based on the preliminary study, a single dose of either 1.2 mg or 1.0 mg SI/0.05 mL was also injected (1.2 mg in n = 5 canines, 1.0 mg in n = 2 canines). Spectral domain-optical coherence tomography (OCT), electroretinography (ERG), and histological examinations were performed at baseline and following intravitreal injection. In the preliminary study, after a 0.5-mg SI injection and a 1.0-mg SI injection and after two 0.8-mg SI injections, retinal degeneration with retinal thinning was observed on OCT imaging. In the second study, after a single 1.0- or 1.2-mg SI injection, outer retinal degeneration was induced. All eyes showed diffuse outer retinal degeneration on OCT and a loss of both cone and rod responses in ERG. Histological examination also showed the loss of outer retinal layer. Intravitreally injected SI (1.0–1.2 mg) in a vitrectomized canine model induced outer retinal degeneration effectively, and could be evaluated through *in vivo* ophthalmic examination.

## Introduction

Retinal degeneration leads to irreversible and severe vision loss and boasts a significant socioeconomic impact. Therefore, to overcome the problems of retinal degeneration, innovative treatments (e.g., visual prosthetics, and stem cell and gene therapies) have recently been developed by combining biomedical and biotechnological retinal research^1^. Electronic device technology and biomaterials have been developed, therefore, visual prosthetics have been also improved to manage retinal degenerative disorders^2,3^. Recently, visual prosthetics was implanted in humans with retinitis pigmentosa (RP) and choroideremia, although there was still a limit to visual improvement and safety^4,5^. Although restoration of visual field and visual acuity, and improvement in activities of daily life could be obtained by the implantation of visual prosthetics in advanced stage of retinal disorders, resolution of visual signal was still low to distinguish objects^6,7^. Furthermore, the cost of visual prosthetics is very expensive^8^ and surgery of visual prosthetics implantation is difficult and could cause complication such as retinal detachment and dislocation of device^7,9^. RP is one of inherited retinal dystrophies and one of the causes of irreversible vision loss. Initial degeneration due to RP occurs in the photoreceptors, and inner retinal thickness is gradually decreased in advanced-stage RP^10–12^. Choroideremia is a rare inherited retinal dystrophy that clinically manifests with gradual vision loss and progressive retinal degeneration with degeneration of retinal pigment epithelium (RPE) cell and photoreceptor, and choroidal atrophy^13^. Because choroideremia is caused by a single mutation or deletion of the CHM gene, gene therapy is an attractive treatment in choroideremia, and both phase II and phase III clinical trials have been actively conducted recently^13,14^. These two diseases result in a common histologic change, which is the loss of the selective photoreceptor layer, leaving a relatively preserved inner retinal layer^11,15,16^. Therefore, to further develop and refine such treatment modalities so as to enhance therapeutic effectiveness and safety, larger experimental animal models (e.g., monkeys, dogs, pigs, and cats) with retinal degeneration with specific losses of photoreceptors are inevitably needed to simulate RP or choroideremia.

There currently exist some drug-induced animal models of retinal degeneration. However, most drug-induced animal models involve small animals such as mice, rats, or rabbits. In larger animal models of retinal degeneration, most, if not all, are genetically modified animals. Few studies have reported large animal models (e.g., cats, dogs, and pigs) with retinal degeneration induced by drug injection, mainly completed via intravenous injection^17–20^. Systemic drug administration to the experimental animal risks inducing retinal degeneration on both eyes and also reducing general health. There has been no canine model with retinal degeneration induced by intravitreal drug injection to date. The advantage of canine models is the similarity in size of the canine eye to the human eye (an axial length of approximately 22 mm), in addition to the fact that canine models have a higher photoreceptor density compared with rodent models^21,22^.

In the present research, unilateral diffuse homogeneous outer retinal degeneration was attempted to induce by way of intravitreal administration of sodium iodate (SI) following vitrectomy in canines. The primary objective of this study was to elucidate the optimal intravitreal SI dose after vitrectomy necessary to induce diffuse outer retinal degeneration in a canine. The second objective of this study was to evaluate the morphological and physiolocal changes after intravitreally injected SI with the determined dose to induce diffuse outer retinal degeneration.

## Results

### Retinal imaging in the preliminary study of sodium iodate after pars plana vitrectomy

**Figure 1:**
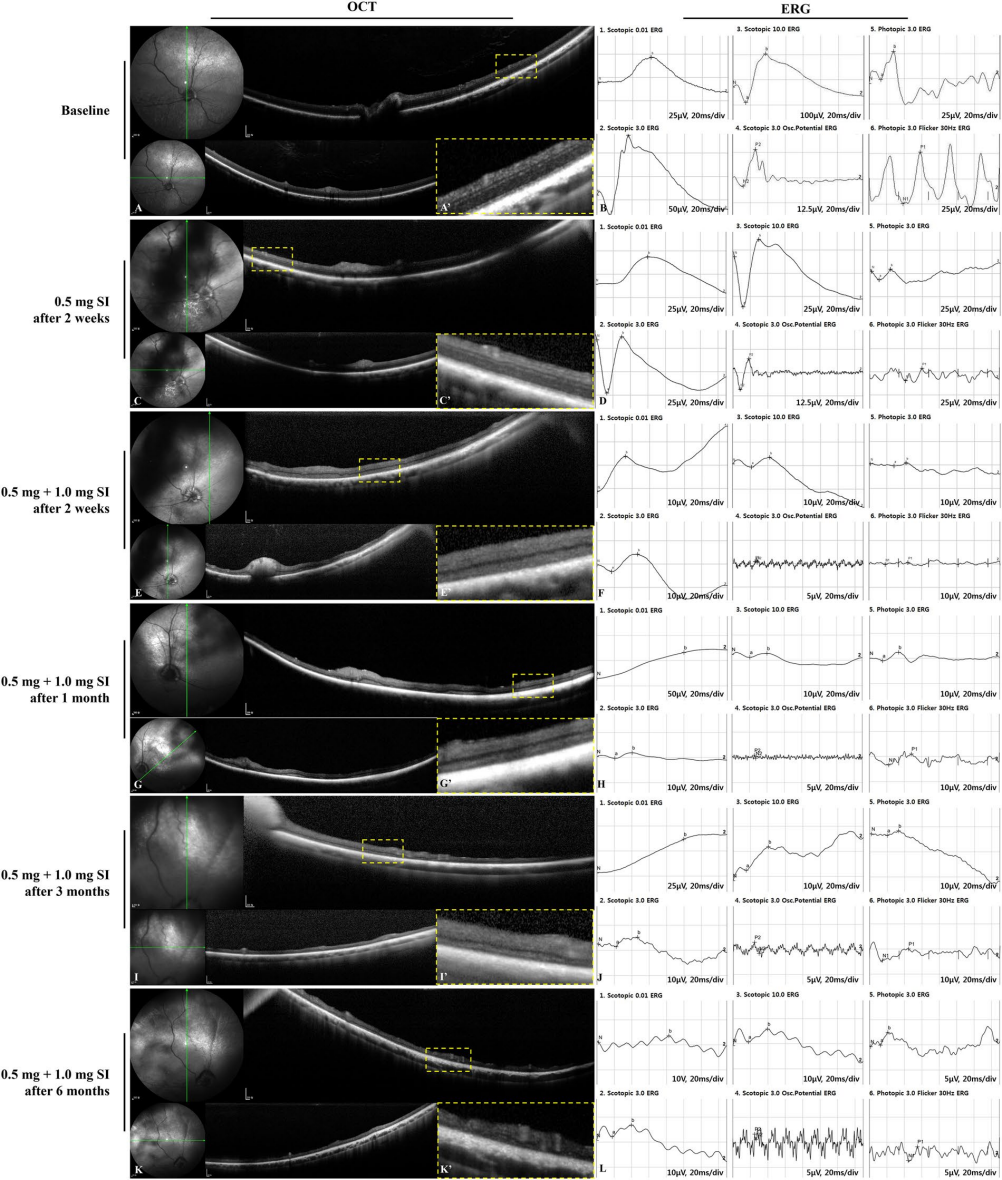
SD-OCT images and ERG data at baseline and after the intravitreal injection of 0.5 mg of SI followed by 1.0 mg of SI in vitrectomized eyes. Compared to baseline, no significant retinal degeneration was observed in OCT, and moderate loss of cones and rods responses in ERG was maintained two weeks after the injection of 0.5 mg of SI (**A**–**D**). After additional administration of 1.0 mg of SI by intravitreal injection in eyes with previous 0.5-mg SI injection, retinal degeneration with retinal thinning in OCT, and a significantly reduced response in ERG were observed at two weeks (**E**,**F**) and these findings were maintained further at one, three, and six months (**G**–**L**). The green line on infrared fundus photography (FP) shows the plane where the SD-OCT images were collected (**A**,**C**,**E**,**G**,**I**,**K**). Magnified SD-OCT images are shown (A’,C’,E’,G’,I’, and K’; dashed-line boxes in Figures **A**,**C**,**E**,**G**,**I**,**K**).

**Figure 2:**
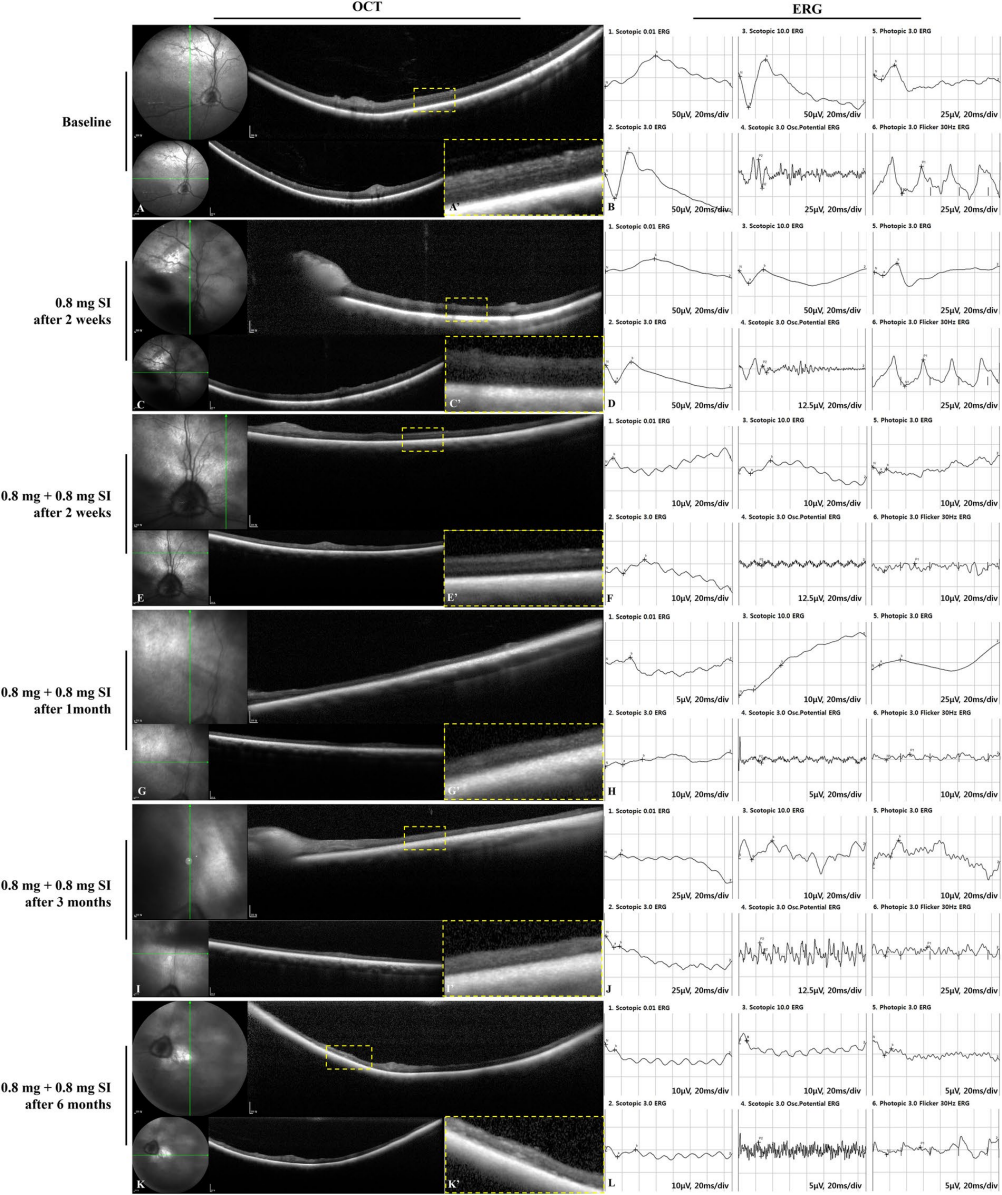
SD-OCT images and ERG data at baseline and after the intravitreal injection of 0.8 mg of SI followed by 0.8 mg of SI in vitrectomized eyes. Compared to baseline, no significant retinal degeneration was observed in OCT, and moderate loss of cones and rods responses in ERG was observed two weeks after the injection of 0.8 mg of SI (**A**–**D**). After repeated administration of 0.8 mg of SI by intravitreal injection in eyes with previous 0.8-mg SI injection, retinal thinning in OCT and a significantly reduced response in ERG were observed at two weeks. (**E**,**F**) Severe retinal atrophy in OCT and abnormal response in ERG were maintained at one, three, and six months (**G**–**L**). The green line on infrared FP shows the plane where the SD-OCT images were collected (**A**,**C**,**E**,**G**,**I**,**K**). Magnified SD-OCT images are shown (A’, C’, E’, G’, I’, and K’; dashed-line boxes in Figures **A**,**C**,**E**,**G**,**I**,**K**).

Compared to the baseline examination, we observed no significant retinal degeneration in spectral domain-optical coherence tomography (SD-OCT) and moderate loss of cone and rod responses in electroretinography (ERG) two weeks after the single administration of either a 0.5-mg or 0.8-mg SI injection (Figs. 1A–D and 2A–D). Retinal degeneration in OCT and reduced response of the cones and rods in ERG were observed two weeks after additional administration of 1.0 mg of SI in the eye with a previous 0.5-mg SI injection and of 0.8 mg of SI in the eye with a previous 0.8-mg SI injection (Figs. 1E,F and 2E,F). Retinal thinning with retinal degeneration in OCT and abnormal responses in ERG were maintained at one, three, and six months (Figs. 1G–L and 2G–L). Although the single injection of 0.5 mg and 0.8 mg of SI did not induce significant retinal degeneration, the intravitreal injection of a repeated or increased dose (0.8 or 1.0 mg of SI) did induce outer retinal degeneration and retinal atrophy with retinal thinning.

### Retinal imaging in the second and the third studies of 1.2-mg or 1.0-mg sodium iodate injection after vitrectomy

**Figure 3:**
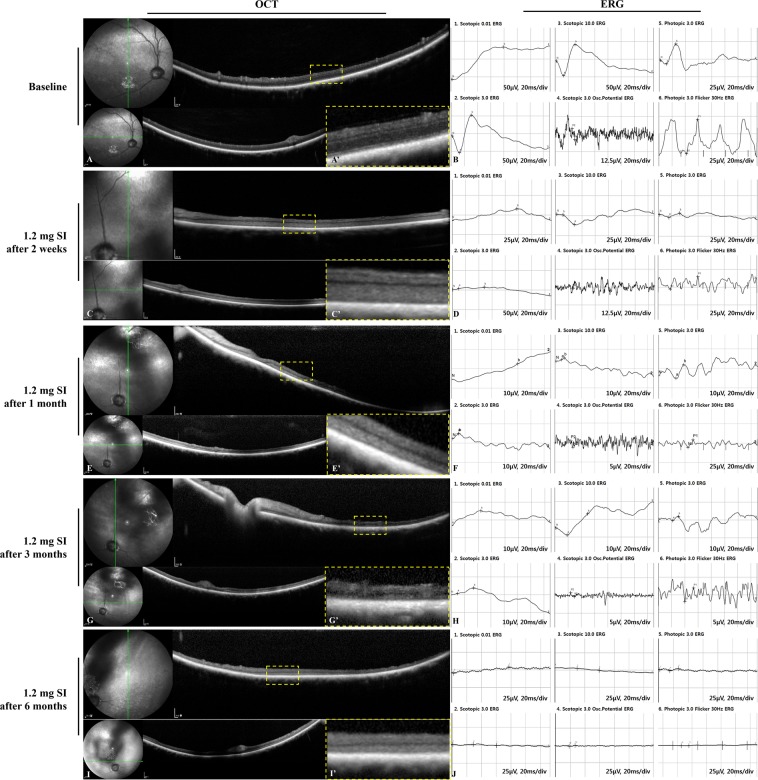
SD-OCT images and ERG data at baseline and after the intravitreal injection of 1.2 mg of SI in vitrectomized eyes. Compared to baseline (**A**,**B**), outer retinal degeneration was observed in OCT, and a reduced response of cones and rods was observed in ERG at two weeks (**C**,**D**), one month (**E**,**F**), three months (**G**,**H**), and six months (**I**,**J**) after the 1.2-mg injection of SI. The green line on infrared FP shows the plane where the SD-OCT images were collected (**A**,**C**,**E**,**G**,**I**). Magnified SD-OCT images are shown (A’, C’, E’, G’, and I’; dashed-line box in Figures **A**,**C**,**E**,**G**,**I**).

**Figure 4:**
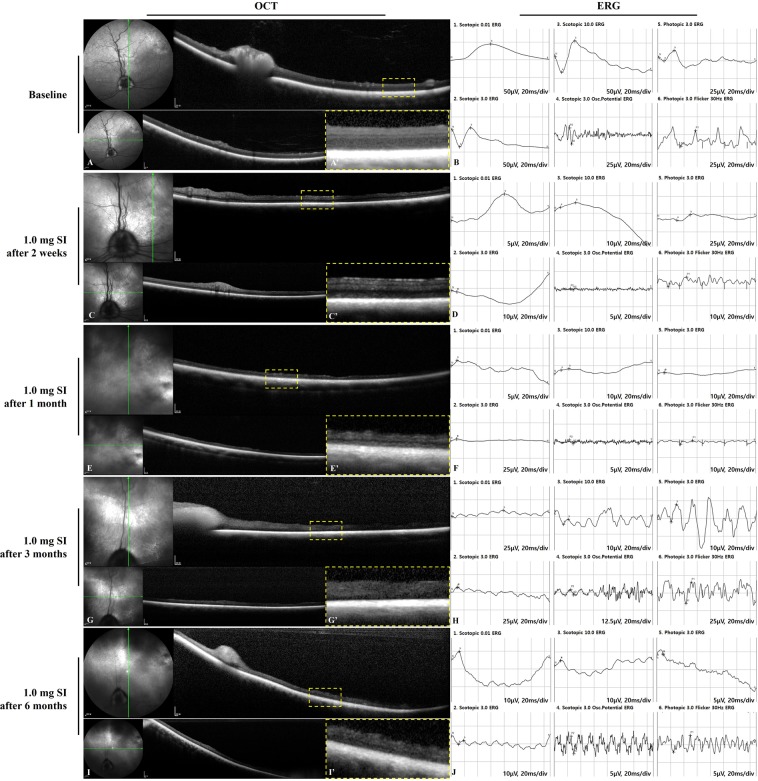
SD-OCT images and ERG data at baseline and after the intravitreal injection of 1.0 mg of SI in vitrectomized eyes. Compared to baseline (**A**,**B**), outer retinal degeneration was observed in OCT, and an abnormal response of cones and rods was observed in ERG at two weeks (**C**,**D**), one month (**E**,**F**), three months (**G**,**H**), and six months (**I**,**J**) after the 1.0-mg injection of SI. The green line on infrared FP shows the plane where the SD-OCT images were collected. (**A**,**C**,**E**,**G**,**I**) Magnified SD-OCT images are shown (A’,C’,E’,G’,I’; dashed-line box in Figures **A**,**C**,**E**,**G**,**I**).

Based on the preliminary study, single doses of 1.2 mg and 1.0 mg of SI in 0.05 mL of total volume were selected for further evaluation. Compared to the baseline examination, outer retinal degeneration including diffuse loss of the IS/OS (inner and outer segment) line and the ONL (outer nuclear layer) was observed in SD-OCT, and the cone and rod responses in ERG were decreased at two weeks after the 1.2-mg SI injection (Fig. 3A–D). These findings were maintained at one month, three months, and six months (Fig. 3E–J). After the 1.0-mg SI injection, the results of SD-OCT and ERG were observed as similar to the results after the 1.2-mg SI injection at two weeks and at one, three, and six months (Fig. 4).

### Retinal degeneration induced by sodium iodate injection after vitrectomy

**Figure 5:**
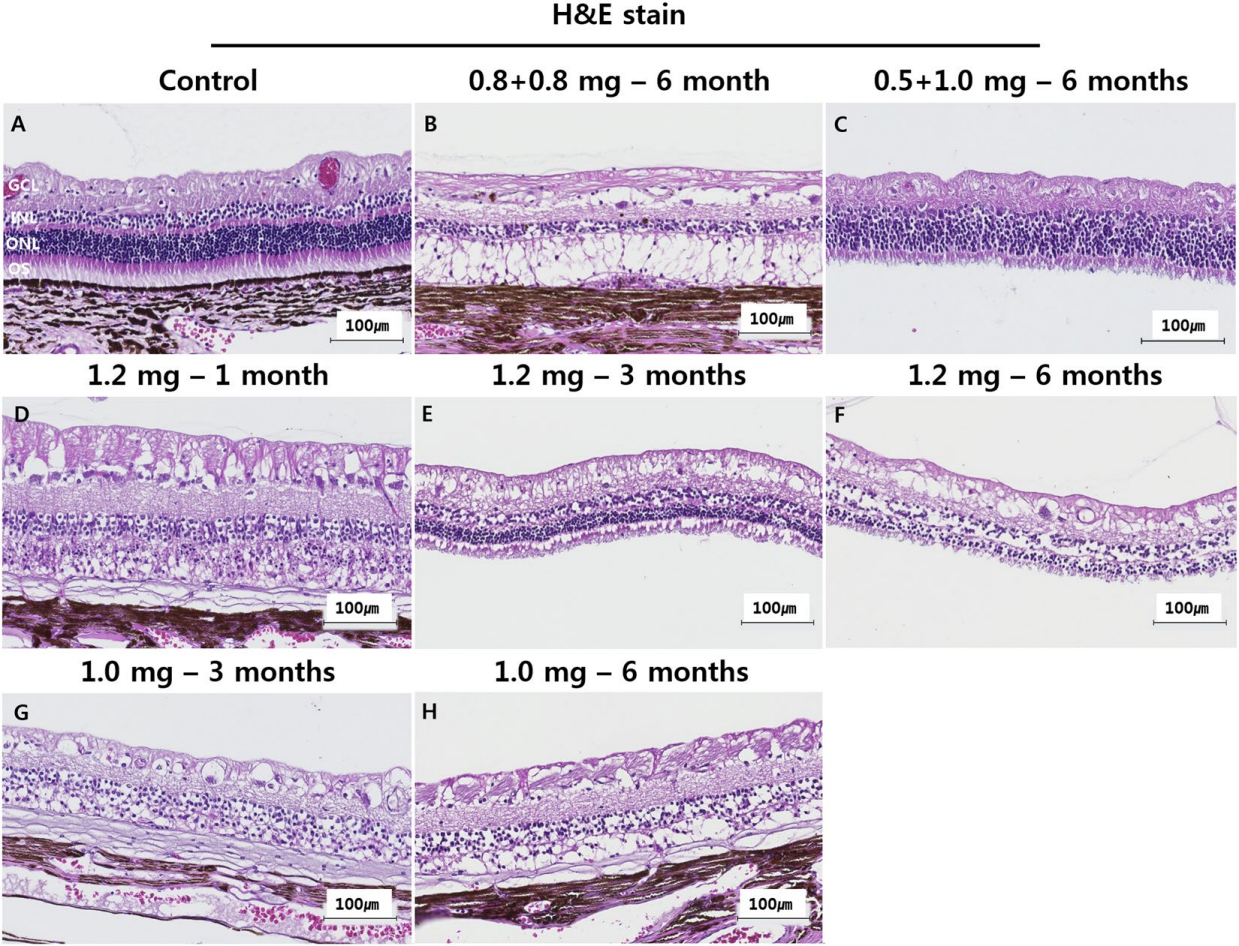
Histological examination by H&E staining after injection with different doses of SI. Histology findings demonstrated the difference between control eyes (**A**) and eyes with retinal degeneration after intravitreal SI injection (**B**–**H**). In control eyes, all layers of the retina were easily distinguished (**A**). Six months after either two 0.8-mg SI injections or 0.5-mg SI injection followed by 1.0-mg SI injection, loss of the photoreceptor layer was observed (**B**,**C**). One, three, and six months after 1.2-mg SI injection, disruption of the photoreceptor layer and thinning of the ONL were observed, while the inner retinal layer remained intact (**D**–**F**). Similar to the case of H&E staining after 1.2-mg SI injection, outer retinal degeneration was shown at three months and six months after 1.0-mg SI injection (**G**,**H**).

An example of histological examination by hematoxylin and eosin (H&E) staining is presented in Fig. 5. In the control eye (fellow eye), all layers of the retina were easily distinguished by histology (Fig. 5A). At six months after two 0.8-mg SI injections, severe retinal thinning with diffuse loss of the ONL and photoreceptor layer was observed, and the inner retina was also slightly destroyed (Fig. 5B). At six months after the 0.5-mg SI injection followed by the 1.0-mg SI injection, H&E staining revealed a minor reduction in the photoreceptor layer, while the inner retinal layer was preserved (Fig. 5C). One month after the 1.2-mg SI injection, disruption of the photoreceptor layer and ONL was observed, while the inner retinal layer remained intact (Fig. 5D). At three months and six months after the 1.2-mg SI injection, H&E staining was similar to the one-month result (Fig. 5E,F). At three months and six months after the 1.0-mg SI injection, outer retinal degeneration was shown by histology with H&E staining (Fig. 5G,H).

**Figure 6:**
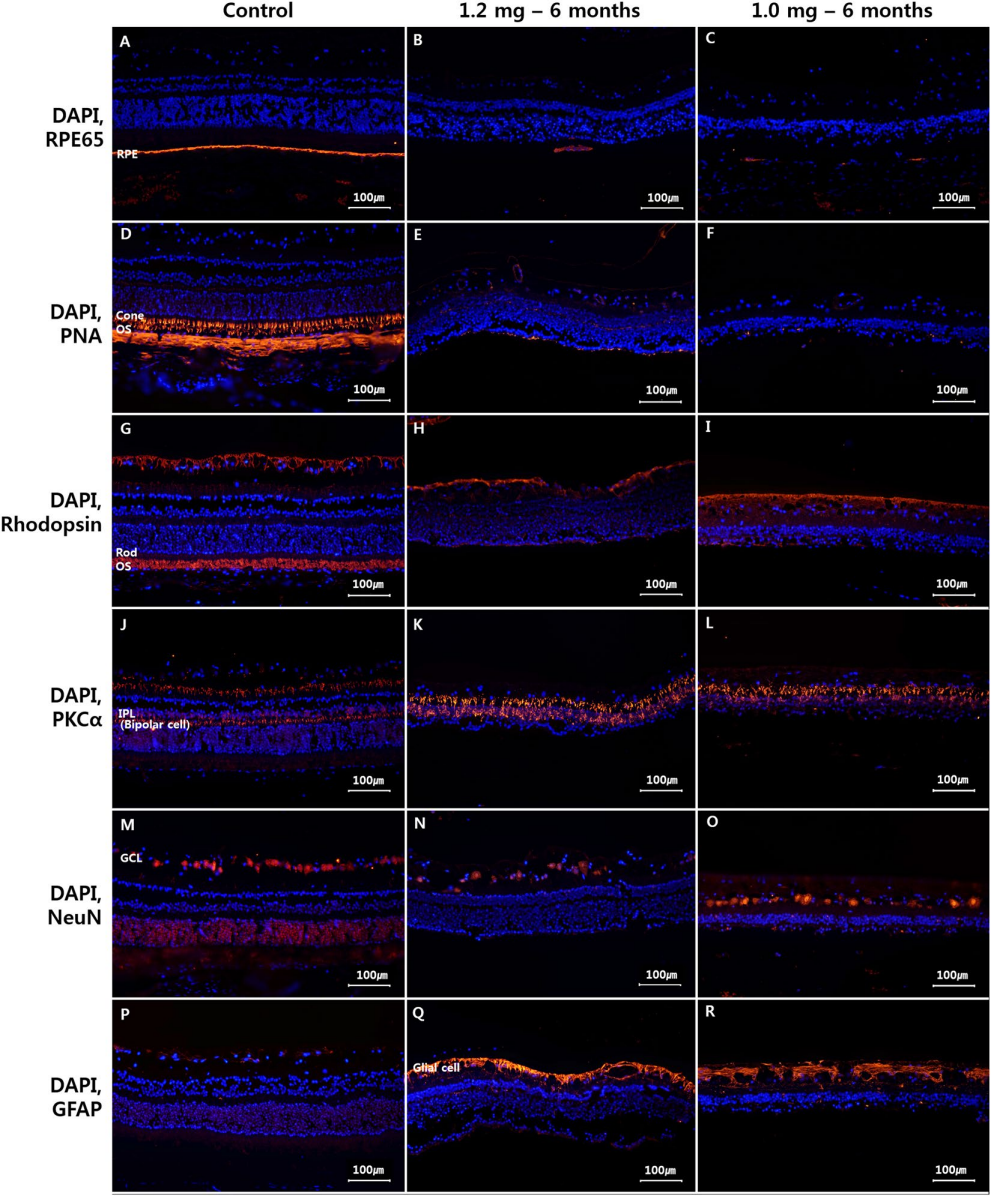
Immunohistochemistry findings at six months after 1.2-mg or 1.0-mg SI injection. In control eyes, the RPE cells, cone and rod photoreceptors, bipolar cells, and ganglion cells were distinctly stained by RPE65, PNA, rhodopsin, PKCα, and NeuN staining, respectively (**A**,**D**,**G**,**J**,**M**). Six months after 1.2-mg SI injection, the expression of the RPE and cone and rod photoreceptors was found to be slightly decreased by RPE65, PNA, and rhodopsin staining (**B**,**E**,**H**), while the numbers of bipolar and ganglion cells were found to be normal based on PKCα and NeuN staining (**K**,**N**). Six months after 1.0-mg SI injection, similar expression of the RPE, cone, rod, bipolar, and ganglion cells was observed as compared with six months after 1.2-mg SI injection (**C**,**F**,**I**,**L**,**O**). When compared with control eyes, the expression of GFAP staining was significantly increased in all of the injected eyes (**Q**,**R**).

Immunohistochemistry findings demonstrated differences between the control eyes and the SI-injected eyes that received 1.2 mg or 1.0 mg (Fig. 6). In the control eyes, the RPE, cone and rod photoreceptors, bipolar cells, and ganglion cells were all easily identified with RPE65, PNA, rhodopsin, PKCα, and NeuN staining, respectively (Fig. 6A,D,G,J,M). In eyes with retinal degeneration after the 1.2-mg or 1.0-mg SI injections, RPE and cone and rod photoreceptor cells were decreased in number based on RPE65, PNA, and rhodopsin staining, respectively (Fig. 6B,C,E,F,H,I). Conversely, the numbers of bipolar cells and ganglion cells were maintained according to PKCα and NeuN staining, respectively (Fig. 6K,L,N,O). Eyes determined to have retinal degeneration after the 1.2-mg or 1.0-mg SI injections demonstrated increased GFAP staining, suggesting a proliferation of glial cells, which was not observed in the control eyes (Fig. 6P–R).

### Quantitative change after 1.2-mg and 1.0-mg sodium iodate injection

We analyzed the effects of a 1.2-mg and 1.0-mg SI injection after vitrectomy.

**Figure 7:**
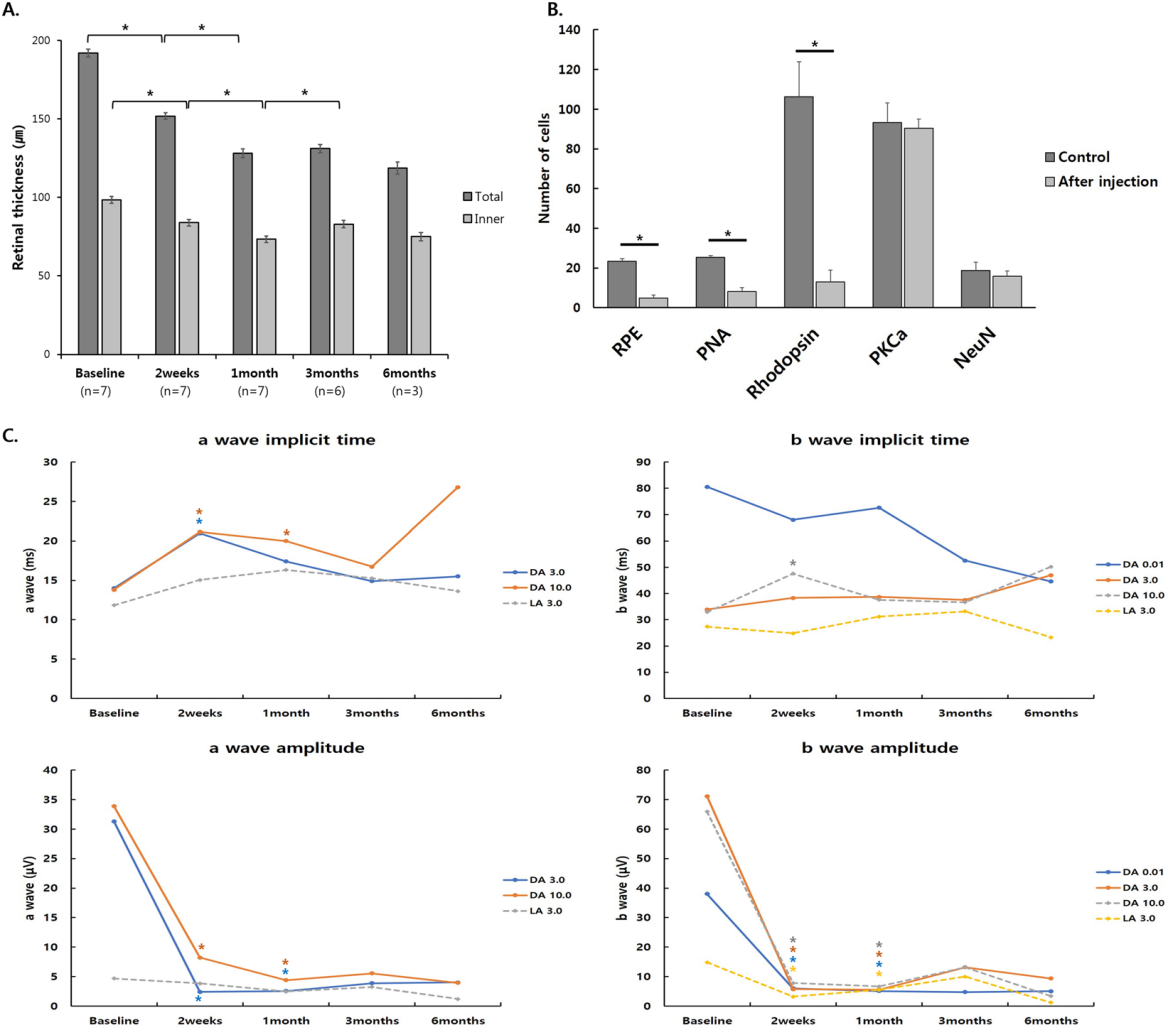
Quantitative changes after 1.2 mg and 1.0 mg SI injection. (**A**) Changes in retinal thickness. Total retinal thickness and inner retinal thickness were significantly decreased at two weeks, one month, three months, and six months after injection. Total retinal thickness was relatively maintained after one month. Inner retinal thickness was relatively maintained after three months. (**B**) Changes in immunohistochemistry. Six months after 1.2 mg and 1.0 mg SI injection, the expression of the RPE and cone and rod photoreceptors was found to be significantly decreased compared to control eyes, while the numbers of bipolar and ganglion cells were not significantly changed. (**C**) Changes in ERG response. After injection of 1.2 mg of SI and 1.0 mg of SI, the amplitude of the a and b waves in scotopic and photopic responses were significantly decreased except the amplitude of the a wave in the photopic 3.0 response. The severe changes in amplitude of the a-wave and b-wave were maintained at 3 months and 6 months. Implicit time of the a-wave in scotopic 3.0 and 10.0 responses at 2 weeks and the b-wave in the scotopic 10.0 response at 2 weeks were significantly delayed. The asterisk indicates a significant difference (*p < 0.05).

Total retinal thickness was significantly decreased after injection (191.93 ± 2.56 μm at baseline vs. 151.76 ± 2.08 μm at two weeks, 128.11 ± 2.66 μm at one month, 131.07 ± 2.59 μm at three months, and 118.67 ± 3.95 μm at six months after injection; p < 0.01, respectively) (two weeks vs. one month, three months, and six months; p < 0.01, respectively) (Fig. 7A). Total retinal thickness was not significantly different from one month to six months after injection (one month vs. three months: p = 0.051; one month vs. six months: p = 0.382; three months vs. six months: p = 0.1). Inner retinal thickness was also significantly diminished up to one month after injection (98.33 ± 2.25 μm at baseline vs. 83.88 ± 2.13 μm at two weeks and 73.31 ± 2.01 μm at one month after injection; p < 0.01, respectively) (Fig. 7A). Three months after injection, inner retinal thickness was significantly different from one month (82.88 ± 2.36 μm at three months; p < 0.01) and was maintained until six months (74.97 ± 2.70 μm at six months; p = 0.114; Fig. 7A).

Six months after 1.2 mg and 1.0 mg SI injection, the expression of the RPE and cone and rod photoreceptors was found to be significantly decreased compared to control eyes (23.33 ± 1.33, 25.33 ± 0.88, 106.33 ± 17.46 in control vs. 4.82 ± 1.58, 8.0 ± 2.01, 13.0 ± 5.96 at six months after injection, respectively; p < 0.01, respectively), while the numbers of bipolar and ganglion cells were not significantly changed (93.33 ± 9.91, 18.67 ± 4.26 in controls vs. 90.36 ± 4.63, 15.73 ± 2.81 at six months after injection, respectively; p = 0.769 and p = 0.659, respectively) (Fig. 7B).

Compared with baseline and after injection of 1.2 mg of SI and 1.0 mg of SI, the amplitude of a-wave and b-wave in scotopic and photopic responses showed severe changes at 2 weeks and 1 month (baseline vs. 2 weeks for the scotopic response; p = 0.018, baseline vs. 1 month for the scotopic response; p = 0.018, baseline vs. 2 weeks for the photopic response; p = 0.018, baseline vs. 1 month for the photopic response; p = 0.043) except the amplitude of the a-wave in the photopic 3.0 response (baseline vs. 2 weeks; p = 0.237, baseline vs. 1 month; p = 0.176) (Fig. 7C). The severe changes in amplitude of the a-wave and b-wave were maintained at 3 months and 6 months (Fig. 7C). Compared to baseline, the implicit time of the a-wave in the scotopic 3.0 and 10.0 responses at 2 weeks (p = 0.028, respectively) and the b-wave in the scotopic 10.0 response at 2 weeks (p = 0.018) was significantly delayed (Fig. 7C).

## Discussion

In this study, we analyzed the effectiveness of SI injection after vitrectomy to induce unilateral diffuse outer retinal degeneration in canine. 1.2 or 1.0 mg of SI in 0.05 mL of total volume was determined to be most effective and optimal dose to induce retinal degeneration, with a disruption of cone and rod photoreceptors after intravitreal injection.

SI as a retinotoxin is an oxidizing compound that is directly toxic to RPE cells and secondarily toxic to photoreceptors and the choroid^23^. Specifically, reactive oxygen species (ROS) is produced by administration of SI and ROS contributes to primarily damage and induce necrosis in RPE cells^24–26^. As change to RPE cells and photoreceptors, SI also provokes choriocapillaris atrophy^27,28^, necrosis of the inner retina^24,29^, and panretinal degeneration^24,30^. It has been reported that various doses and routes of administration of SI cause retinal toxicity in many different experimental animals (e.g., sheep^25^, rabbits^25,31^, rats^30,32^, and mice^23,29,33^). Most studies have employed relatively high doses of SI (50–100 mg/kg) and have reported rapid RPE damage characterized by the defragmentation and loss of RPE cell nuclei. Systemic administration of SI provokes retinal degeneration on both eyes and also makes general health worse in the experimental animals. Systemic intoxication after systemic administration of SI has been reported to not only cause gastrointestinal problems, general weakness, and convulsion and but also fatality in experimental animals^34,35^. Therefore, local administration of SI is warranted in order to avoid its systemic toxicity. In the present study, intravitreal SI injection in canine eyes induced outer retinal degeneration with loss of photoreceptor and RPE cells, and these effects were observed in SD-OCT, ERG, and histology with H&E staining and immunohistochemistry. Furthermore, there were no significant systemic complications.

It was difficult to predict the correct dose of SI with intravitreal injection in different animals to induce retinal degeneration. The effectiveness of vitrectomy was reported in our previous study when N-methyl-N-nitrosourea (MNU) was intravitreally injected to lead retinal degeneration^36^. In that study, when vitrectomy was not performed in rabbit eye, diffuse outer retinal degeneration by intravitreal injection of MNU was not induced. Based on these past results, we also attempted to produce a drug-induced rabbit model with outer retinal degeneration caused by intravitreal injection of SI following vitrectomy^37^. We found that an intravitreal 0.4 mg/0.05 mL SI injection after vitrectomy produced diffuse outer retinal degeneration with damage to the photoreceptors in rabbit eyes. In the present study, we analyzed retinal degeneration in canine eyes induced by the intravitreal injection of 1.0 or 1.2 mg of SI after vitrectomy. In our previous study, the mean axial length of the rabbit eyes was actually measured as 15.96 mm, while, in our present study, the mean axial length of the canine eyes was measured as 21.50 ± 0.42 mm, as similarly reported in prior literature^21,38^. Thus, the difference in axial lengths between rabbits and canines was about 1.4-fold; if converting to volume, the difference in volumes between the two was measured as about 2.74 times. Therefore, the dose of SI for intravitreal injection after vitrectomy was predicted to be about 1.1 mg, or the value obtained by multiplying 0.4 mg of SI by 2.74, and the predicted dose of SI was approximately matched with the real experimental results as 1.0 to 1.2 mg of SI. Based on our previous and present studies using two different species of animals, vitrectomy could help to induce diffuse and relatively homogenous retinal degeneration and, when vitrectomy is performed, the dose of SI that induces retinal degeneration could approximately predict axial length.

Few studies have explored large animal models with retinal degeneration induced by drug injection mainly done through intravenous injection. Intravenous iodoacetic acid injection in cats and swine generated retinal degeneration^18,20^. Although intravenous SI injection in swine did not provoke definite retinal degeneration, retinal degeneration was induced by local subretinal injection with SI in swine^17^. In canines, drug-induced retinal degeneration was found following intravenous SI injection^19^. To the best of our knowledge, there is no prior canine model of retinal degeneration induced by intravitreal drug injection in existence, so the present study is the first article presenting a drug-induced canine model with retinal degeneration through localized administration. Besides, in the present study, the long-term results at six months were also evaluated to determine the effectiveness of intravitreal SI injection in canine eyes. For six months, retinal degeneration induced by a 1.0 mg or 1.2 mg injection of SI was presented as an anatomical change that was confirmed by SD-OCT and histology with H&E staining and immunohistochemistry and a functional change that was confirmed by ERG.

Notably, there were some limitations in this study that should be mentioned. First, the number of animals included was relatively small. Furthermore, to analyze the longitudinal change for six months, some of the animals were sacrificed sequentially at regular intervals for six months. Therefore, to evaluate the quantitative changes in retinal thickness, immunohistochemistry and ERG response, we combined both 1.2 mg and 1.0 mg SI injection results. However, with the number of canines included, we were able to decide the effective dose and evaluate the effects of intravitreal SI injection after vitrectomy on the induction of diffuse retinal degeneration. Thus, while minimizing the number of sacrificed animals, the aim of this study could be achieved. Second, the accuracy of ERG could be affected by anesthetic agents, especially isoflurane. As reported in some studies, significant attenuation and delay of the ERG response were observed after anesthesia and sedation in animal models^39^. The ERG response also varied according to different anesthetic agents in animal models^40–42^. In particular, isoflurane can decrease b-wave amplitude and implicit time in light-adapted ERG and dark-adapted ERG more so than other anesthetic agents^40,43^. However, during the period from baseline to six months after injection, general anesthesia in canines was performed according to the same anesthetic protocol by the same veterinarian doctor in the same experimental environment. Therefore, there is thought to be no effect of general anesthesia on the change in ERG results.

In conclusion, a vitrectomized canine model subjected to an intravitreal 1.0- or 1.2-mg/0.05-mL SI injection induced diffuse outer retinal degeneration with disruption of photoreceptors. Canine models with outer retinal degeneration could be useful as a large animal model for further research into innovative treatments.

## Materials and Methods

### Animals

All procedures adhered to the Association for Research in Vision and Ophthalmology (ARVO) Statement for the Use of Animals in Ophthalmic and Vision Research (ARVO Animal Policy). Approval for this study was obtained from the Institutional Animal Care and Use Committee of the Korea University College of Medicine in Seoul, Korea and all procedures were performed in accordance with the relevant guidelines and regulations.

Eyes randomly selected in female mixed breed dogs (n = 10) received an intravitreal injection of SI two weeks after vitrectomy. The canines had a mean body weight of 33.08 ± 3.07 kg, and their mean axial length according to A-scan ultrasonography was 21.50 ± 0.42 mm. For all SI injections, each dose of SI was diluted in 0.05 mL of balanced salt solution (BSS; Alcon, Fort Worth, TX, USA).

The principle behind choosing the number of study subjects in this study was to use the minimal number of animals required. There was no literature to refer to in choosing the candidate dose for intravitreal injection in canines or any other large animals except our previous study^37^, in which an intravitreal 0.4 mg/0.05 mL SI injection after vitrectomy induced diffuse outer retinal degeneration with a disruption of photoreceptors in rabbit eyes. The vitreous volume has been known to be about 1.5 mL in rabbit eyes and about 2.9 mL in canine eyes, according to previously published literature^38,44^. Thus, the difference in vitreous volume between the two animals is about twofold. Therefore, the present study was designed as follows: injections of 0.5 mg/0.05 mL (n = 2) and 0.8 mg/0.05 mL (n = 1) were attempted first. If these tested doses did not successfully induce retinal degeneration in the canine eye, then a double dose (1.0 mg/0.05 mL) was injected again in the 0.5 mg/0.05 mL case, and the same dose (0.8 mg/0.05 mL) was injected again in the 0.8 mg/0.05 mL case (Supplementary Fig. S1A). Based on the preliminary study, in the second complementary study, a 1.2 mg/0.05 mL SI dose was selected, which was injected into the main study group in this study (n = 5) (Supplementary Fig. S1B). A third complementary study was also conducted, where a 1.0 mg/0.05 mL SI dose was injected (n = 2) (Supplementary Fig. S1B).

To identify morphological changes to the retina, SD-OCT with infrared reflectance (IR) imaging was performed at baseline and two weeks after intravitreal SI injection in the preliminary study. To identify physiological changes to the retina, ERG was also performed at baseline and two weeks after injection. After the second doses were injected, SD-OCT and ERG were repeatedly performed two weeks, one month, three months, and six months after injection. Histological examinations after H&E staining were also completed six months after the second injection.

In the complementary studies, SD-OCT and ERG were performed at baseline, two weeks, one month, three months, and six months after injection, while histological examinations after H&E staining were performed one month, three months, and six months after the 1.2 mg SI injection or three and six months after the 1.0 mg SI injection.

### Vitrectomy

All canines were routinely premedicated and anesthetized. Premedication consisted of the subcutaneous injection of atropine (0.05 mg/kg, Je-Il atropine sulfate®; Je-Il pharmacy, Daegu, Korea) and intramuscular injection of xylazine (1 mg/kg, Rompun®; Bayer AG, Leverkusen, Germany), and anesthetic induction was conducted by the intravenous injection of alfaxalone (1–2 mg/kg, Alfaxan®; Jurox Pty. Ltd.; Rutherford, Australia). Canines were intubated and maintained under general anesthesia via the inhalation of 1.5 to 2.5% isoflurane (Ifran®; Hana Pharm Co., Ltd.; Gyeonggi-do, Korea) delivered in 50–70% oxygen with a flow rate of 2 L/min.

After general anesthesia, 0.5% tropicamide and 0.5% phenylephrine (Tropherine®; Hanmi Pharm Co., Ltd., Seoul, Korea) were administered for pupil dilatation, and then the eye was irrigated with 5% povidone iodide and draped for surgery. Sclerotomy for 23-gauge trocar cannula insertion was done with a 20-gauge needle because of the rigid canine sclera (Supplementary Fig. S2A). The three ports were prepared by inserting a trocar cannula into the sclera 4 mm from the limbus on the superior side within 150 degrees of the meridian, because a large nictitating membrane under the lower eyelid limited the surgical area able to be used for placing trocars (Supplementary Fig. S2B). Three-port, 23-gauge core vitrectomy (Associate; DORC, Zuidland, the Netherlands) was performed with a direct plano-concave and meniscus lens (Hoya, Tokyo, Japan). The vitreous was removed using a vitreous cutter while continually supplying BSS.

### Intravitreal injection of sodium iodate

Animals were anesthetized as described above. Immediately before the injections, SI (Sigma-Aldrich, St. Louis, MO, USA) was dissolved in BSS. The eye of each canine was prepared, and the corresponding dose of SI (in a total volume of 0.05 mL) was injected intravitreally at 4 mm posterior to the limbus using a 30-gauge needle. No injections were performed in the fellow eyes.

### Spectral-domain optical coherence tomography

SD-OCT was performed using the Spectralis® OCT system (Heidelberg Engineering GmbH, Heidelberg, Germany)^36^. The retinal area near the optic disc was evaluated. Vertical line scans, horizontal line scans, and raster scans (33 B-scans over a 16.5-mm × 16.5-mm area in a 55-degree image) were performed in high-resolution mode (1,536 A-scans per B-scan, lateral resolution = 10 µm/pixel in a 55-degree image). Up to 100 single images were averaged in the automatic real-time mode to obtain a high-quality mean image.

### Electroretinography

The ERG protocol was based on the international standard for ERG from the International Society for Clinical Electrophysiology of Vision (ISCEV) and previous our studies^19,36,37,45^. The dogs were anesthetized as described above, dark-adapted for 30 minutes, and their pupils dilated. One eye per animal was studied in order to avoid accidental contralateral light adaptation. Light stimulation and ERG signal recording were performed with a commercial system (RETIcom; Roland Consult, Brandenburg an der Havel, Germany) using a contact lens electrode with a built-in light source (Kooijman/Damhof ERG lens; Medical Workshop BV, Groningen, the Netherlands). The reference and ground electrodes were platinum subdermal needle electrodes. The reference electrodes were placed in the skin near the lateral canthus of the eyes, while the ground electrode was placed on the forehead between the two eyes.

The a-wave amplitude was measured from the baseline to the trough of the first negative wave; the b-wave amplitude was measured from the trough of the a-wave to the peak of the first positive wave or, if the a-wave was absent, from baseline to the peak of the first positive wave^27^.

### Histological examination

Immediately after euthanasia, eyes were enucleated, immersion-fixed in Davidson’s solution for 24 hours, dehydrated, and embedded in paraffin. Sections measuring 4 µm were cut and stained with H&E. The slides were examined for pathological changes in the retina using a light microscope (BX-53; Olympus Corp., Tokyo, Japan) and photographed with a digital camera (INFINITY3-1UR; Lumenera Corp., Ottawa, ON, Canada)^36^.

### Immunohistochemistry

Tissue sections were deparaffinized, rehydrated, and microwave-heated in antigen retrieval buffer (1 mM of EDTA, 0.05% Tween 20, pH: 8.0). Sections were then blocked with 4% horse serum in phosphate-buffered saline (PBS), which was followed by primary antibody incubation at 4 °C overnight. Anti-RPE65 (Invitrogen, Carlsbad, CA, USA) staining was performed following the manufacturer’s protocol. For anti-PKCα (Invitrogen, Carlsbad, CA, USA) and rhodamine-labeled anti-peanut agglutinin (PNA) (Vector Laboratories, Burlingame, CA, USA) coimmunostaining, fluorescence detection was performed with an Alexa Fluor 488–conjugated goat anti-mouse secondary antibody (Invitrogen, Carlsbad, CA, USA). For anti-PKCα and anti-rhodopsin (Rockland Immunochemicals, Pottstown, PA, USA) coimmunostaining, fluorescence detection was performed with Alexa Fluor 488–conjugated goat anti-mouse and Alexa Fluor 594-conjugated goat anti-mouse secondary antibodies (Invitrogen, Carlsbad, CA, USA). Anti-neuronal nuclei (NeuN) (Chemicon, Temecula, CA, USA) staining was performed following the manufacturer’s protocol^36^. For anti-GFAP (Novus Biological, Littleton, CO, USA) staining, sections were incubated for two hours at room temperature and then for one hour with Alexa Fluor 594-conjugated goat anti-mouse secondary antibody. Nuclei were counterstained with 4,6-diamidino-2-phenylindole (DAPI) (AnaSpec Inc., Fremont, CA, USA). Cells with staining were evaluated using fluorescence microscopy (T2000-U; Nikon, Tokyo, Japan)^37^.

To quantify the expression of cells with RPE65, PNA, rhodopsin and PKCα, the number of cells within the 200 μm range was automatically counted using ImageJ software (version 1.51; National Institutes of Health, Bethesda, MD, USA). To quantify the expression of cells with NeuN, the number of cells within the image total range was also counted.

### Retinal thickness measurement for retinal thinning induced by sodium iodate injection

In each canine, we measured the total retinal thickness and inner retinal thickness at 10 different retinal sites using linear horizontal SD-OCT imaging performed at baseline and at two weeks, one month, three months, and six months after a 1.2-mg SI/0.05-mL and 1.0 mg SI/0.05 mL injection as mentioned in the previous our study^37^. Total retinal layer was defined as from the ganglion cell layer (GCL) to the RPE layer, and inner retinal layer was defined as from the GCL to the inner nuclear layer (INL).

### Statistical analysis

To compare the effects of SI injection on number of cell counts in immunohistochemistry from control to post-injection, statistical analysis was conducted using a Mann-Whitney test. To compare the effects of SI injection on total retinal thickness and inner retinal thickness from baseline to post-injection, statistical analysis was conducted using a Wilcoxon signed-rank test. Statistical analysis was also conducted using a Wilcoxon signed-rank test to compare the effects of SI injection on implicit time and amplitude of the a-wave and b-wave in the scotopic and photopic responses of ERG from baseline to two weeks, one month, three months, and six months after a 1.2 mg SI/0.05 mL and 1.0 mg SI/0.05 mL injection. Data are presented as mean ± standard error. Differences were considered statistically significant at p < 0.05.

## Supplementary information

Supplementary Figure S1, Supplementary Figure S2.
