# Supplementary material for: Morphologic and electrophysiologic findings of retinal degeneration after intravitreal sodium iodate injection following vitrectomy in canines
Source: Sci Rep. 2020 Feb 27;10:3588. doi: 10.1038/s41598-020-60579-1 (PMC7046695; doi:10.1038/s41598-020-60579-1)

**Morphologic and electrophysiologic findings of retinal degeneration after intravitreal sodium iodate injection following vitrectomy in canines**

So Min Ahn, MD^1^; Jungryul Ahn^2^; Seongkwang Cha^2^; Cheolmin Yun, MD, PhD ^1^; Tae Kwann Park, MD, PhD^3^; Young-Jin Kim^4^; Yong Sook Goo, MD, PhD^2^; and Seong-Woo Kim, MD, PhD^1*^

^1^Department of Ophthalmology, Korea University College of Medicine, Seoul, Korea

^2^Department of Physiology, Chungbuk National University School of Medicine, Cheongju, Korea

^3^Department of Ophthalmology, Bucheon Hospital, Soonchunhyang University College of Medicine, Bucheon, Korea

^4^Medical Device Development Center, Osong Medical Innovation Foundation, Cheongju, Korea

**Supplementary Figure S1.** Injection schedule.

A. Injection schedule in the preliminary study.

B. Injection schedule in the second and the third complementary study.


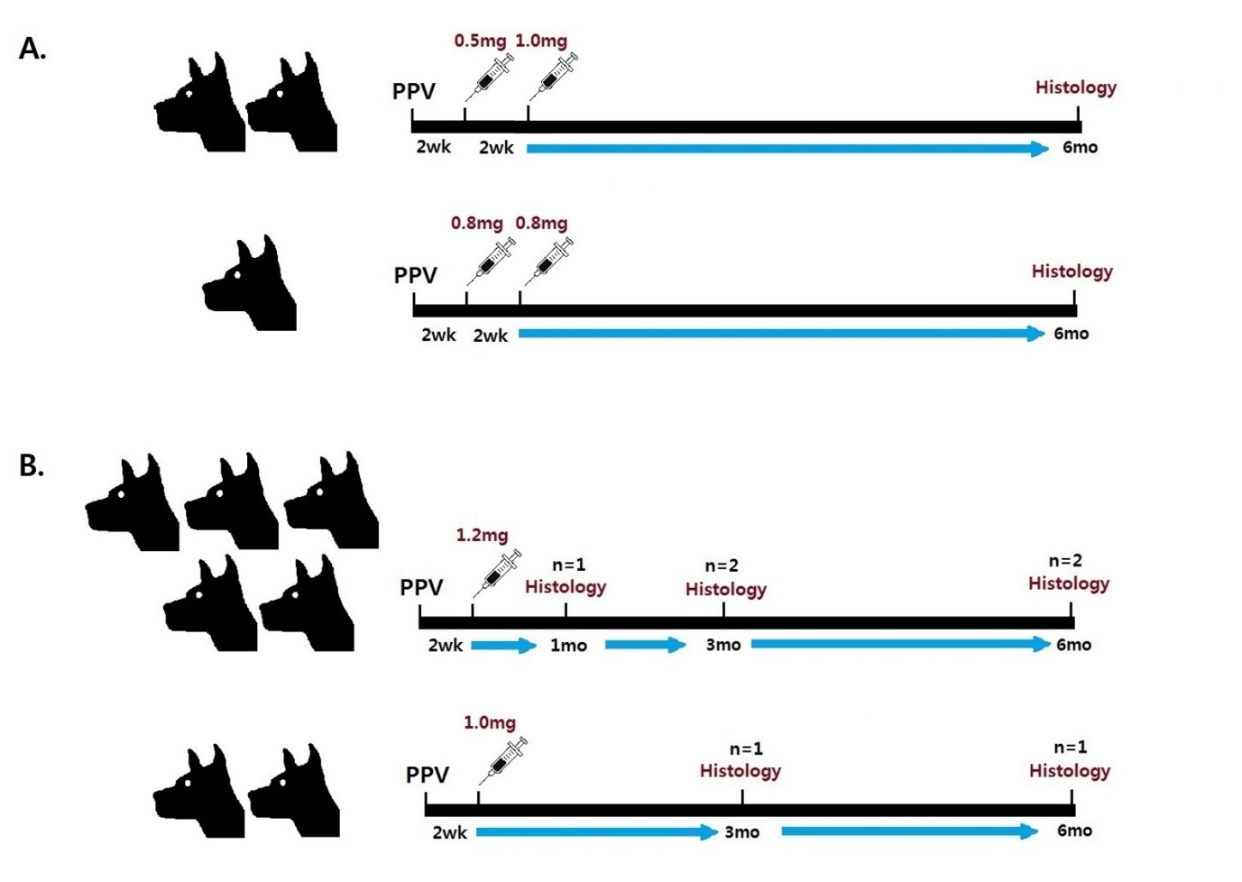


**Supplementary Figure S2.** Procedure for 23-gauge vitrectomy in the canine model.

A. Sclerotomy with a 20-gauge needle 4 mm from the limbus.

B. After insertion of a three-port 23-gauge trocar in superior (dorsal) side of the eye.


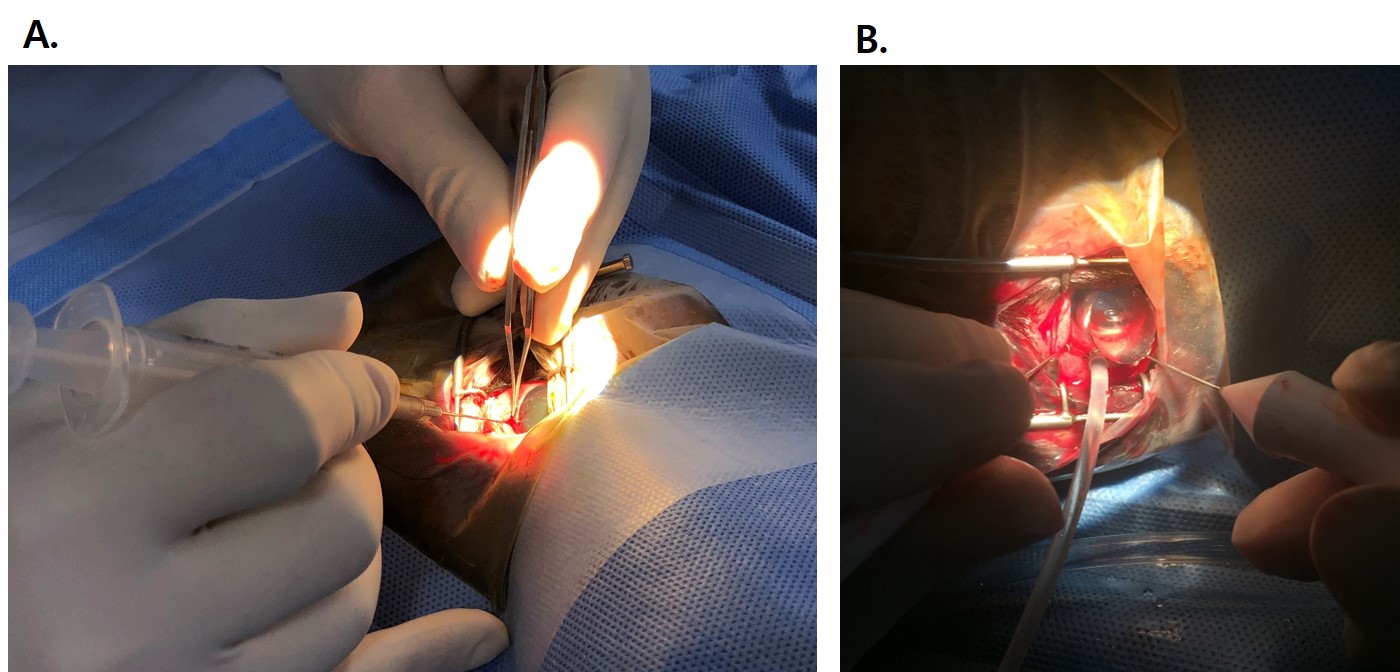

Supplement: Supplementary file 1 — Supplementary Figure S1, Supplementary Figure S2. [file 41598_2020_60579_MOESM1_ESM.docx]
